# Honeybees express foodward flight vectors after a detour

**DOI:** 10.1242/jeb.251072

**Published:** 2025-11-20

**Authors:** Anna Hadjitofi, Marie Messerich, Tim Landgraf, Barbara Webb

**Affiliations:** ^1^ School of Informatics, University of Edinburgh, Edinburgh EH8 9AB, UK; ^2^ Dahlem Center for Machine Learning and Robotics, Freie Universität Berlin, 14195 Berlin, Germany

**Keywords:** Honeybees, Vector navigation, Waggle dance, Detour experiment

## Abstract

The honeybee waggle dance communicates a flight vector to a food source, but it is challenging to isolate how precisely dancers and recruits can navigate using this vector information independently of environmental cues. We introduce an enforced-detour paradigm, using tunnels, to quantify the initial flight vectors expressed by experienced foragers and new recruits en route to the food. Upon exiting the detour, bees exhibit immediate corrective turns consistent with using path integration to fly towards the food's virtual location. While the populations' flight bearings after the turn are correctly centred on the food, the bearings of individuals are considerably scattered around it. We further show that recruits' bearings can be predicted by observing their mechanical sensory experiences during dance following. Our findings suggest that the communicated or recalled vector can be combined with path integration to take corrective shortcuts, but also that the vector provides an approximate location rather than pinpoint accuracy.

## Teaser

**Summary:** Honeybees can combine their recalled or communicated flight vector with path integration to make immediate corrective turns towards food after navigating a detour at the beginning of the journey.

## INTRODUCTION

After a successful foraging trip, a honeybee has internal knowledge of the flight vector to the food, which she can communicate by producing a stereotyped motor pattern in the hive, known as the waggle dance. This creates mechanical cues that surrounding nestmates (follower bees) can assimilate to obtain their own flight vector, gaining knowledge of the distance and direction of food relative to the hive that they have not themselves directly experienced. Previously, the behavioural expression of these vectors has been measured by tracking flight paths using harmonic radar (e.g. Riley et al., 2005; Wang et al., 2023); recording the arrival of bees at traps around the feeder location (e.g. Johnson, 1967; Gould, 1975; Tanner and Visscher, 2008); and observing vanishing bearings, where the angle of a bee's departure from a location is measured by eye (e.g. Dyer, 1991; Chittka et al., 1995, Menzel et al., 2000).

Early work portrayed the dance as a precise language; assuming recruits could pinpoint the food location based on the dance vector alone (Munz, 2005). For example, von Frisch (1967) observed dance recruits arriving within ±15 deg of the advertised feeder. Subsequent work has shown that recruits can likely supplement their vector with other cues to pinpoint the resource, including interactions with other bees (Tautz and Sandeman, 2003; Tautz, 2022), scents (Griffin et al., 2012) and knowledge of the environment (Wang et al., 2023). The integration of multiple information sources makes it difficult to isolate path integration and estimate the accuracy afforded by the dance vector alone. A recent review by Egelhaaf and Lindemann (2025) argues that whilst path integration is robustly evidenced in walking insects, experimentally isolating its navigational use in flying insects (beyond its expression in the dance) is considerably more challenging. Whether the dance vector is compared, in flight, to the bee's own path integration state, enabling it to take shortcuts to the vector location independently of environmental cues, is considered by these authors to be an open question. Some evidence that suggests this capability may exist is the observation of recruits performing novel flights between a dance-indicated location and a previously learned feeder location without guiding landmarks (Menzel et al., 2011).

Tactile input to the antennae has long been considered to play a role in how nestmates interpret the dance (Rohrseitz and Tautz, 1999; Gil and De Marco, 2010), but the underlying mechanism was unclear. We recently proposed a neural model showing how a bee could recover a vector by combining its antennal displacement with its orientation to gravity (Hadjitofi and Webb, 2024). Using real antennal position data of dance followers as input, the simulated circuit predicted a distribution of the recruits' vectors that is correctly centred on the food direction, but with substantial variance (±90 deg around it). This motivated us to experimentally measure how accurately bees use vector information alone to fly toward a resource.

In this paper, we introduce a new methodology to estimate honeybees' flight vectors as they navigate to a food source. Inspired by the enforced-detour paradigm in ant navigation (Schmidt et al., 1992; Collett et al., 1999), we use narrow tunnels to impose an initial detour on bees leaving the hive and measure their corrective angle upon release to get a readout of their intended vector, isolated from environmental cues. Tunnels have been used to manipulate dancer signalling and the returns of foragers to feeders (Srinivasan et al., 2000; Esch et al., 2001, Si et al., 2003, De Marco and Menzel, 2005; Dacke and Srinivasan, 2007; Shafir and Barron, 2010), but we believe this is the first report of using tunnels to manipulate recruit behaviour. We compare the individual post-detour vector directions to the vectors expressed in dances by individual foragers and to the vectors we predict (from our model) to have been assimilated by individual followers.

## MATERIALS AND METHODS

### Experimental protocol

#### Study site

**Fig. 1.**
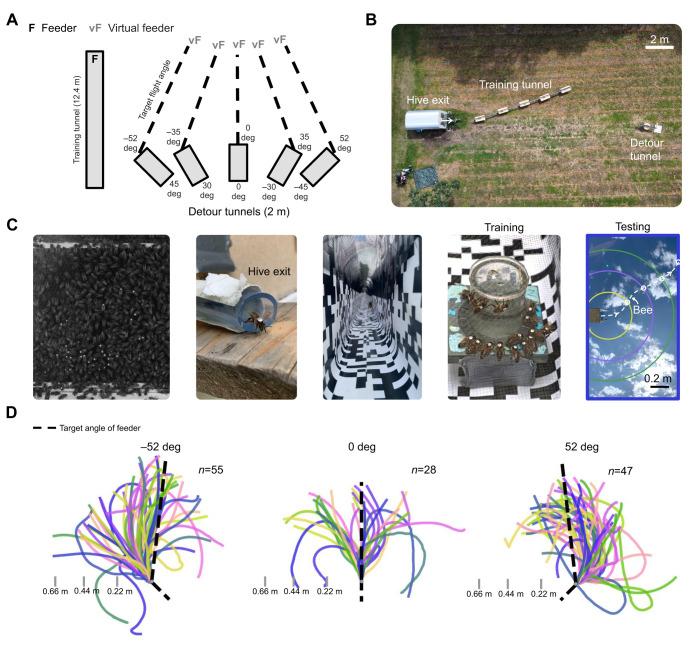
**A detour experiment in honeybees.** (A) Relative configurations of tunnels. F and vF respectively indicate the feeder location during training and its target virtual location after a detour. Sizes and angles not drawn to scale. (B) Top-down view of the experimental area. (C) Experimental procedure. After training, experienced foragers or new recruits leaving the hive (presumably to fly their foodward vector) are displaced to a different tunnel that imposes a detour at the start of their journey. Their angle of flight post-detour is recorded at boundaries from the exit: 0.22 m (yellow), 0.44 m (purple), 0.66 m (green) and last visible position (blue). (D) Top-down view of foragers' raw post-detour flight trajectories for relative detour angles of 45, 0 or −45 deg.

The study site was located in the grounds of Julius Kühn-Institut Berlin, in the district of Dahlem (Germany). A small colony of *Apis mellifera carnica* Pollman 1879 was housed in a two-frame observation hive within a trailer positioned at one end of the field (Fig. 1B). The colony had been moved from their previous home near Freie Universität Berlin to the site 1 month prior to the experiment. The data were collected between 6 June and 27 July, 2023. Prior to the study, bees were regularly captured as they left the hive and marked with circular numbered tags to later identify them in the experiment.

#### Tunnels

Tunnels were used to control the bees' foraging routes and experience during the experiment. They were assembled daily from rectangular segments that were each 2.6 m long, 9 cm wide and 26 cm high. The side walls and floor were lined with waterproof paper that displayed a random white and black Julesz pattern, with each block being 1 cm by 1 cm. Flying past these patterns exploits the bees' visually gauged odometer and causes them to perceive that they have flown a much farther distance (i.e. several hundred metres) than they have (i.e. 6 m), which is accordingly signalled in the dance (Srinivasan et al., 2000). Moreover, this non-periodic pattern prevents the use of cues based on counting a sequence of landmarks or features. A fine black mesh was used as a roof to allow light into the tunnel and a view of the sky, so that the bees could still perceive celestial directional cues (Rossel and Wehner, 1984). The mesh was affixed at mid-height to prevent the bees from flying near the top and viewing nearby landmarks (see also Menzel and Galizia, 2024).

#### Training

The first step was to establish a feeder location – a target vector – that foragers would be trained to and subsequently signal in their dances. A group of bees were thus trained to a feeder (1:1 sugar water) inside a training tunnel whose entrance was 3 m from the hive. This mirrored the setup of previously published tunnel experiments (Srinivasan et al., 2000; Esch et al., 2001). The feeder was initially placed at the entrance, before being gradually moved further inside until it was at the end (12.4 m). The tunnel was closed at this end so that the bees would fly the same route to and from the feeder. The final distance of the feeder remained constant after training. The orientation of the tunnel relative to the hive was also constant within a given day but changed across days to be either 0 deg or ±20 deg. Based on the observation that the accuracy of flight trajectories remained consistent across these feeder angles (Fig. S1A), we pooled the data for the main analyses. We recorded the identities of bees visiting the feeder frequently (every 5 min) and filmed their dances in the hive at 80 frames s^−1^ under 640 nm red light. Training continued until at least ten foragers were consistently visiting the feeder at its final position and signalling it in their dances.

#### Testing

At this point, foragers would frequently revisit the hive to advertise their vector to the feeder before returning to it, and new recruits who had just followed a dance would now attempt to fly their newly acquired vector to the feeder for the first time. In the testing phase, we attempted to capture these bees immediately as they exited the hive and displaced them to the entrance of a different tunnel that forced the first segment of their outbound journey to be a 2 m detour, i.e. potentially forcing them to fly in a different direction to the actual hive–food direction. This tunnel was open at the far end to allow the bees to exit and make any corrective turn necessary to intersect their estimated location of the food, assuming they were comparing their current location estimated by path integration during the detour to their hive–food vector, obtained from either a previous foraging trip or dance following. A 60 frames s^−1^ camera positioned beneath the tunnel recorded the silhouette of the bee against the sky as she left the detour. The camera field of view covered a 1.56 m long and 0.84 m wide horizontal plane at the tunnel's height, 1 m above the ground. Assuming bees make a corrective turn, there are two possible predictions for the size of the correction: that they turn to the bearing of the dance vector (e.g. a −45 deg turn after a 45 deg detour); or that they turn to take the shortcut direction towards the target food location (e.g. a −52 deg turn after a 45 deg detour of 2 m from a 12.4 m flight path) (Fig. 1A). To obtain the vector difference between the bee's actual flight path and the target shortcut vector, the azimuth of the target vector (from the tunnel exit straight to the virtual feeder) was calculated using the time that the bee was released into the detour, the date and the location of the hive, and assumed that the length of the vector corresponding to flight within the tunnel was determined by the physical dimensions of the detour.

Initial testing of the foragers revealed that detour angles that were too extreme relative to the virtual feeder (e.g. ±60 deg and ±90 deg) often resulted in bees refusing to fly the detour altogether. Instead, they would fly back and forth near their release point in the tunnel with their bodies oriented approximately towards the virtual feeder location. Therefore, to promote a consistent motivation to fly the detour, we used a range of smaller detour angles (±45 deg and ±30 deg for foragers, and ±30 deg for recruits), as well as a ‘detour’ of 0 deg to provide a condition where no corrective turn would be necessary. We excluded from later analysis the data from any bee that stopped flying within the tunnel or took longer than 30 s to exit. We also categorised the sky conditions during each flight and found that their post-detour accuracy did not differ significantly across the conditions (Fig. S1B,C, Mardia–Watson–Wheeler test: *W*_6_=11.222, *P*=0.082). During testing, the training tunnel remained accessible to the other foragers, so as to maintain a continuous supply of experienced foragers and recruits for testing in the detour tunnel.

### Data analysis

#### Tracking post-detour flights

**Fig. 2.**
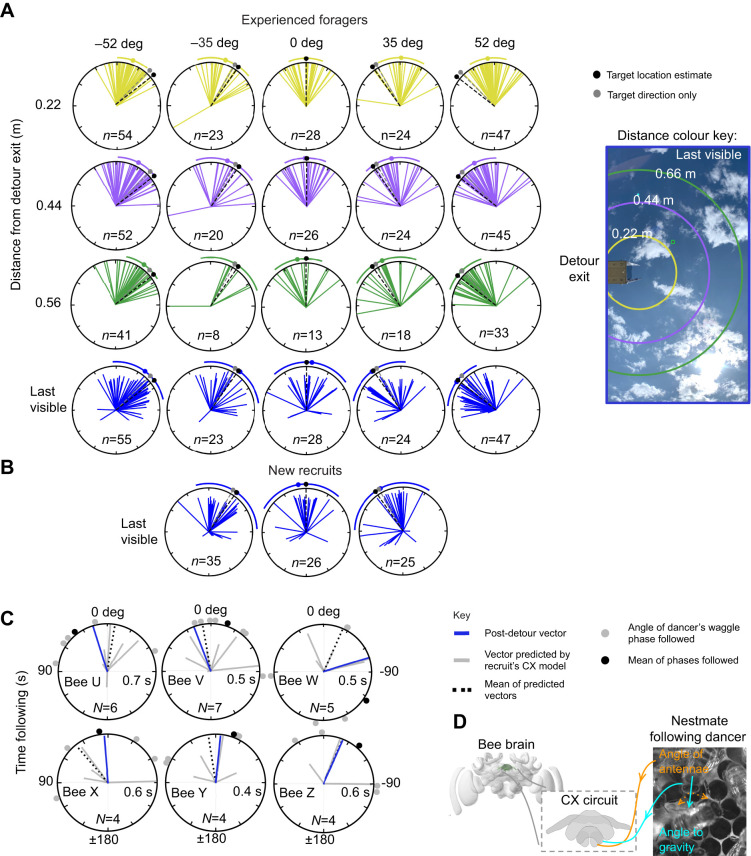
**Distributions of post-detour flight angles for experienced foragers (dancers) and new recruits (follower bees).** (A,B) Columns indicate target angle required to reach the feeder upon exiting a detour and coloured rows indicate the distance boundary from the detour exit. Target directions shown for navigating based on the feeder's location (black) or its direction only (grey). Coloured dots and arcs indicate circular means±s.d. (C) The vectors of six new recruits predicted by the assimilation circuit at the end of each waggle phase (lines), the angle of phases they followed (circles) and their subsequent angle of flight post-detour (blue) relative to the feeder. (D) Schematic of the assimilation circuit proposed by Hadjitofi and Webb (2024), which models the central complex (CX) in the insect brain (shown in green in a frontal view of the bumblebee brain, obtained from www.insectbraindb.org).

**Fig. 3.**
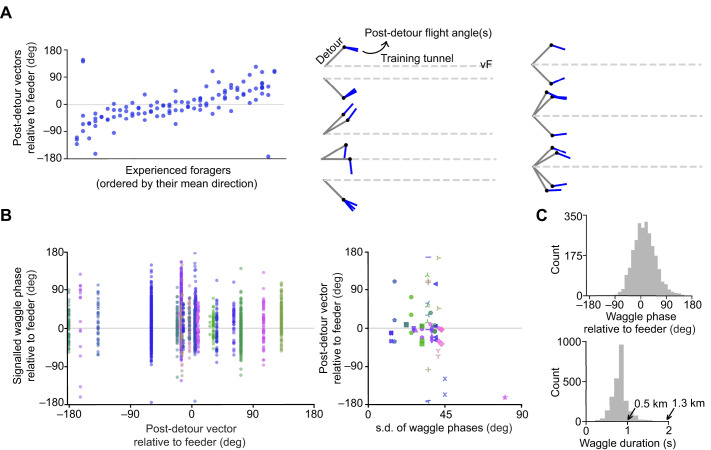
**Individual forager performance.** (A) Post-detour angles for individual foragers (*n*=33) tested repeatedly on the same detour angles relative to the feeder (‘vF’) within the same day. Examples of individuals tested on different detour angles are shown on right. Vertically stacked points in the scatter plot belong to the same individual. (B) All foodward angles from dancers (*n*=24) plotted against their post-detour accuracy (mean direction, left) and precision (s.d., right). Each coloured marker represents an individual. (C) Histogram of waggle phase angles and durations from all dances in B. Arrows indicate equivalent feeder distances in the natural environment (Kohl and Rutschmann, 2021).

Bees were automatically tracked in the videos of their detour tunnel exits using a custom Python script that made use of the OpenCV library and a custom-built GUI (available at https://github.com/annahadji/bee-flight-tracer). After selecting the first visible position of the bee, a Gaussian mixture-based background subtraction algorithm was applied to the video to isolate the bee from the background as it moved (Zivkovic, 2004). The centre Cartesian coordinates of the nearest contour to the bee's position in the previous frame was selected to construct the trajectory. The trajectories were reliably tracked in almost all cases because the bees left the field of view in less than a second (0.8±0.36 s; mean±s.d.), making them the only set of moving pixels within that time. In the few cases where wind caused other objects to move, the bee's position was manually tracked. The angle of the straight line connecting the coordinates of the bee in each frame to its position when first exiting the tunnel was calculated relative to the angle of the detour, which had been normalised to East in the recorded videos. To date, post-detour adjustments have primarily been studied in walking animals (Collett and Collett, 2000), where it may be possible to observe the full length of the trajectory and/or where the corrective turning point may be easier to discretely pinpoint. To determine where this post-detour adjustment would be most evident for a bee in flight, the angles were noted at four boundaries: three concentric circles representing 0.22 m, 0.44 m and 0.66 m from the detour exit on the tunnel's horizontal plane, as well as the last visible position of the bee in the video. Figs 1D, 2 and 3A visualise these trajectories or angles from a top-down view. Under normal circumstances, bees tend to fly upwards when travelling outwards from their hive. We observed that the mesh roof of the tunnel meant that the bees generally became accustomed to flying at that height as they emerged and most were seen to make only a gradual ascent as they departed from the camera view into the natural environment.

#### Measuring flight speed in the detour

The mean flight speed of foragers within the detour tunnel was calculated from video recordings of a random sample of 30 foragers flying through the tunnel. The time taken to travel 0.2 m along a portion of the midsection of the tunnel was measured to the nearest 0.016 s. This flight data was recorded from the midsection of the tunnel to avoid the initial acceleration phase of the bee.

#### Measuring the precision of dances

The angle of a dancer's waggle phase in a video recording was estimated using the straight line connecting their thorax in the first to the last frame that waggling was seen (Fig. 3B,C) or that a nestmate was following (Fig. 2C). This is a standard method for estimating the angle (Arra et al., 2025), although note that this may result in more variance than averaging the dancer's body orientation (Landgraf et al., 2011). A nestmate was defined as following a dance if facing towards the dancer and within one bees-length. The dance angles were manually labelled with respect to gravity on the comb and transformed to be relative to the true angle of the feeder by calculating the expected angle of the feeder on the comb based on the sun's position at the time and date that the dance had been performed. The duration of waggle phases was measured to the nearest 0.05 s (Fig. 3C).

#### Assimilation circuit predictions

For each follower bee that was recorded interacting with a dancer trained to the feeder, the angle of their heading relative to gravity and the position of their antennae were measured in every frame. The position of each antenna was estimated as the angle from its base to its tip, and the midpoint between the left and right antennae was then calculated. These data (specifically, the midpoint of the antennae angles and the angle to gravity) was fed directly into a simulation of the neural circuit described in Hadjitofi and Webb (2024), which produces an estimate of the vector that the follower could assimilate from its actual sensory experience. We visualise the vector from each waggle phase the follower experienced as well as the mean vector direction calculated across consecutive phases they followed (Fig. 2C). We can compare this to the angles directly measured from video of the dancer (see previous section above) for the same set of phases, which to date has been the standard method used to estimate (from a human observer's point of view) what information could have been communicated to the follower.

#### Angles

Any comparisons between angles are presented such that a positive error angle indicates a deviation, in degrees, to the left of the target angle (counterclockwise rotation) and a negative error indicates a deviation to the right (clockwise). Equal Kappa tests for the homogeneity of concentration parameters were used to compare whether the spread of the distributions of final flight angles differed between dancers and followers for the same target angles.

## RESULTS AND DISCUSSION

### Estimating the intended vector from post-detour flight

We inferred a bee's intended vector from her initial post-detour flight (within 2 m of the detour exit) to capture its bearing before subsequent environmental cues might alter its expression (Fig. 1). We first examined foragers assumed to be returning to the training feeder, taking their prior repeated visits to it as an indication they had (1) formed a vector memory to its location, (2) would express this location in their dances, and (3) would also express it in their next flight. Fig. 1A–C shows the experimental setup and Fig. 1D shows examples of the foragers' raw flight trajectories observed post-detour. We calculated a bee's angle relative to the detour exit as she crossed three successive boundaries, as well as her final visible angle before leaving the camera's field of view.

For foragers experiencing detours, Fig. 2A reveals a progressive shift in the distribution of their position across each boundary. The final visible angle (blue) shows the largest shift and is strikingly centred on the virtual feeder location, yet with a wide distribution. Notably, as soon as the path is unconstrained (yellow), there is a bias towards the virtual feeder. Flying bees appear to display a similar tendency to walking insects of immediately initiating a corrective turn, consistent with a path integration control system immediately steering towards the direction that minimises the angle between the current and desired headings (Mittelstaedt, 1962; Stone et al., 2017). However, as there is only a small angular separation between the desired heading that would be predicted from the shortcut direction to the virtual feeder, versus resuming the original vector bearing, we cannot distinguish here which possibility better accounts for the data (but see below). The fact that distributions for the 0 deg distance-only ‘detour’ are also centred on the feeder confirms the observed shifts are not simply an urge to circle away after exiting the tunnel. The consistent correction towards the feeder direction, despite different relative angles of detour, indicates that this paradigm is effective at eliciting a reasonable and directed response across the bees. We thus consider the final visible angle of a bee in our data to be a reliable indicator of the bee's intended foodward vector.

Bees appear to fly slower in the tunnels than in their natural environment (averaging 0.44 ms^−1^, versus 6 ms^−1^ reported by Wenner, 1963). This reduced speed is consistent with our intended manipulation of increased optic flow (by patterning the tunnels) and probably facilitated the corrective flight adjustments being made shortly after the detour exit, within our recording zone. On average, bees left the camera's view in just 0.8±0.36 s, which would also curtail the immediate use of external environmental cues for localisation.

### Vectors of recruited bees

Similarly to experienced foragers (Fig. 2A), newly recruited bees show a wide distribution of final bearings, but one that is strikingly centred on the expected target vector required to intersect the feeder (Fig. 2B). While the overall concentration of the two distributions shows no statistically significant difference, a number of recruits appear to deviate substantially from the expected angle (i.e. >±90 deg). Our data include all bees that flew sensibly along the detour, regardless of their subsequent angle of post-detour flight, so might also include those that emerged from the tunnel with a reduced motivation for foraging. Similar alternative behaviours, such as initiating a homing flight instead of continuing towards the food after a displacement, have been described in other studies of honeybees (Wang et al., 2023) and ants (Collett and Collett, 2000).

### Dance-following experience predicts subsequent behaviour

The clear centring of flight bearings on the virtual feeder, and the wide scattering around this direction, are both consistent with our previous model of the vector information that recruits could obtain from their experience when following dances within the hive. For a sample of six recruits in the current experiment, we were able to record their complete antennal position data for every waggle phase that they followed before attempting to fly their new vector to the feeder. We fed this data into our neural model to obtain predictions of the vectors that they would be expected to recover from each phase of the dances they followed (Hadjitofi and Webb, 2024).

Fig. 2C shows that the real vectors expressed by these recruits after the detour fall within the range of the mean of the assimilated vectors predicted by the neural model (Fig. 2D). Moreover, whilst only for a small sample size, the real vectors correspond more closely to the model's vectors than to the angles calculated directly from measurement of the dancer's angle in the corresponding waggle phases (e.g. bees ‘V’, ‘W’, ‘Y’ and ‘Z’). Thus, although experimenters have historically predicted recruits' search flights from the angles of the dancers' waggle phases, recruits might be able to obtain a better vector estimate from their experience of the dance, as revealed by their antennal positioning. In fact, this is the first demonstration that it is possible to obtain an estimate of the foodward vector that has been communicated to a recruit solely from observing her own behaviour (specifically, the angle of her antennae and her own orientation to gravity) when following a dance in the hive.

We found that there was no specific bias towards recruits expressing a vector from any particular phase in the dance sequence (Fig. S2), supporting the idea that recruits may average or weight information from multiple dance circuits to refine their estimate (Tanner and Visscher, 2008).

### Repeated testing of individual foragers reveals a consistent vector

Surprisingly, foragers returning to a familiar site showed a spread of vectors almost as large as that for new recruits. To examine this further, we analysed the path of foragers who had been captured and tested several times in the detour condition. Many of these individuals exhibited flight directions that were more consistent than the overall population variation might have suggested (Fig. 3A, left). This is also a good indicator that they were indeed attempting to navigate to a specific location after the detour. Some individual foragers were tested with several different relative detour angles. Despite this, each individual's flight paths appeared to converge to a distinct location (Fig. 3A, right). This supports the conclusion that the bees were able to use their foodward vector and their current path integration estimate of their location after the detour to correct their path to a particular target location (not just to a compass direction), and that the experimental method was capturing a bee's intended navigational goal.

### Dance precision is not related to vector accuracy

We then questioned whether the wide distribution of foragers' vectors, as expressed after the detour, was reflected in their dances. Do foragers with more accurate vectors signal the foodward direction with greater precision? We analysed video recordings of the dance sessions for a random sample of foragers before they were caught and tested in the detour (*n*=24). Fig. 3B reveals that the directions signalled in waggle phases were widely scattered around the feeder direction, with no apparent relationship between the precision of dance signalling and the dancer's vector accuracy after the detour. Nearly all dancers signalled, in at least one or two phases, directions that deviated from the target by more than ±60 deg. It thus appears there is a degree of scatter present in dance communication that is consistent across dancers, regardless of how accurately their inferred vector estimates the feeder location. A similar observation was obtained when comparing the duration of waggle phases across these dances, which signal the distance to the feeder (von Frisch, 1967) (Fig. S3). Histograms of the population data are shown in Fig. 3C. This also suggests that the recruits might have been navigating to the resource as best as they could, given the inherent variability in the information available from dancers.

### Conclusions

In this study, we applied an enforced-detour paradigm using tunnels to isolate and quantify the initial in-flight expression of honeybees' foodward vectors. Both experienced foragers and new recruits, when forced into a detour upon leaving the hive, made immediate corrective turns consistent with using a path integration system to fly towards the virtual location of the feeder. The very act of isolating vector-based navigation in experimental settings presents a challenge: while displacement to a test area is necessary to remove familiar cues, it may introduce navigational artifacts due to the disruption of displacement itself (Collett et al., 1999). Our approach mitigates this by observing only the immediate turn of the bee as she emerges from the detour; and that for experienced foragers, the detour itself provides a visual experience highly similar to their training route. As a consequence, our experimental paradigm offers a unique perspective into the precision of navigational vectors.

Our findings also relate to the path integration capabilities of flying insects, as reviewed by Egelhaaf and Lindemann (2025) and suggest that bees can initiate shortcuts based solely on path integration following a displacement, as there was no immediate landscape cue in our experiment that could account for them making the particular corrective turns. Our preliminary tests revealed that bees were reluctant to fly detours that deviated more than 60 deg. As a result, the configurations we used had a relatively small difference between the target angle expected if the bees were aligning with the compass direction of the feeder from the nest, versus the direction from the detour exit, i.e. the shortcut to the feeder location. The converging angles for bees experiencing different detours suggest the latter, but more examples would be needed to establish this. Future adaptations, including longer detours, could also be used to dissociate these possibilities, although this might again increase the difficulty to induce bees to fly the detour.

For a small, but high quality, sample of data points, we were also able to uniquely relate a recruit's intended foodward vector to her own positional information when following a dance (specifically, the displacement of her antennae and her heading relative to gravity; see Hadjitofi and Webb, 2024). The results show that nestmates can recover an approximate vector from the dance that is useful for orienting towards the goal, consistent with the first phase of the recruitment model proposed by Tautz (2022). However, with such a broad scattering of vectors, it appears surprising how well recruits, in past research, have been able to find the goal, even when it is a small saucer of sugar water in a field. It is possible that a structured search based on their vector, along with other orientation aids, could be used in the final stages of locating the resource. For example, Reynolds et al. (2007) reported that honeybees' search loops can deviate 30 to 50 m from their flight vector, and Menzel and Greggers (2013) suggest that bees could be redirected by an odour within an angle that deviates from their vector by up to 60 deg. Thus, the recovered dance vector need not pinpoint the exact food location but nonetheless, can guide recruits closer to it than would be possible if no other cue is available.

## Supplementary Material

10.1242/jexbio.251072_sup1 Supplementary information

## References

[JEB251072C1] Arra, A., Rutschmann, B. and Kohl, P. L. (2025). Comparison of two methods for decoding honeybee waggle dances. *Apidologie* 56, 47. 10.1007/s13592-025-01164-1

[JEB251072C2] Chittka, L., Kunze, J., Shipman, C. and Buchmann, S. L. (1995). The significance of landmarks for path integration in homing honeybee foragers. *Naturwissenschaften* 82, 341-343. 10.1007/BF01131533

[JEB251072C3] Collett, M. and Collett, T. S. (2000). How do insects use path integration for their navigation? *Biol. Cybern.* 83, 245-259. 10.1007/s004220000168 11007299

[JEB251072C4] Collett, M., Collett, T. S. and Wehner, R. (1999). Calibration of vector navigation in desert ants. *Curr. Biol.* 9, 1031-1034. 10.1016/S0960-9822(99)80451-5 10508615

[JEB251072C5] Dacke, M. and Srinivasan, M. V. (2007). Honeybee navigation: distance estimation in the third dimension. *J. Exp. Biol.* 210, 845-853. 10.1242/jeb.002089 17297144

[JEB251072C6] De Marco, R. and Menzel, R. (2005). Encoding spatial information in the waggle dance. *J. Exp. Biol.* 208, 3885-3894. 10.1242/jeb.01832 16215216

[JEB251072C7] Dyer, F. C. (1991). Bees acquire route-based memories but not cognitive maps in a familiar landscape. *Anim. Behav.* 41, 239-246. 10.1016/S0003-3472(05)80475-0

[JEB251072C8] Egelhaaf, M. and Lindemann, J. P. (2025). Path integration and optic flow in flying insects: a review of current evidence. *J. Comp. .ogy A* 211, 375-401. 10.1007/s00359-025-01734-9 PMC12081508 40053081

[JEB251072C9] Esch, H. E., Zhang, S., Srinivasan, M. V. and Tautz, J. (2001). Honeybee dances communicate distances measured by optic flow. *Nature* 411, 581-583. 10.1038/35079072 11385571

[JEB251072C10] Gil, M. and De Marco, R. J. (2010). Decoding information in the honeybee dance: revisiting the tactile hypothesis. *Anim. Behav.* 80, 887-894. 10.1016/j.anbehav.2010.08.012

[JEB251072C11] Gould, J. L. (1975). Honey bee recruitment: the dance-language controversy: unambiguous experiments show that honey bees use an abstract language for communication. *Science* 189, 685-693. 10.1126/science.1154023 1154023

[JEB251072C12] Griffin, S. R., Smith, M. L. and Seeley, T. D. (2012). Do honeybees use the directional information in round dances to find nearby food sources? *Anim. Behav.* 83, 1319-1324. 10.1016/j.anbehav.2012.03.003

[JEB251072C13] Hadjitofi, A. and Webb, B. (2024). Dynamic antennal positioning allows honeybee followers to decode the dance. *Curr. Biol.* 34, 1772-1779. 10.1016/j.cub.2024.02.045 38479387

[JEB251072C14] Johnson, D. L. (1967). Honey bees: do they use the direction information contained in their dance maneuver? *Science* 155, 844-847. 10.1126/science.155.3764.844 6018197

[JEB251072C15] Kohl, P. L. and Rutschmann, B. (2021). Honey bees communicate distance via non-linear waggle duration functions. *PeerJ* 9, e11187. 10.7717/peerj.11187 33868825 PMC8029670

[JEB251072C16] Landgraf, T., Rojas, R., Nguyen, H., Kriegel, F. and Stettin, K. (2011). Analysis of the waggle dance motion of honeybees for the design of a biomimetic honeybee robot. *PLoS ONE* 6, e21354. 10.1371/journal.pone.0021354 21857906 PMC3153927

[JEB251072C17] Menzel, R. and Galizia, C. G. (2024). Landmark knowledge overrides optic flow in honeybee waggle dance distance estimation. *J. Exp. Biol.* 227, jeb248162. 10.1242/jeb.248162 39319438 PMC11529883

[JEB251072C18] Menzel, R. and Greggers, U. (2013). Guidance by odors in honeybee navigation. *J. Comp. .ogy A* 199, 867-873. 10.1007/s00359-013-0850-6 23974855

[JEB251072C19] Menzel, R., Brandt, R., Gumbert, A., Komischke, B. and Kunze, J. (2000). Two spatial memories for honeybee navigation. *Proc. R. Soc. Lond. B Biol. Sci.* 267, 961-968. 10.1098/rspb.2000.1097 PMC1690634 10874744

[JEB251072C20] Menzel, R., Kirbach, A., Haass, W. D., Fischer, B., Fuchs, J., Koblofsky, M., Lehmann, K., Reiter, L., Meyer, H., Nguyen, H. et al. (2011). A common frame of reference for learned and communicated vectors in honeybee navigation. *Curr. Biol.* 21, 645-650. 10.1016/j.cub.2011.02.039 21474313

[JEB251072C21] Mittelstaedt, H. (1962). Control systems of orientation in insects. *Annu. Rev. Entomol.* 7, 177-198. 10.1146/annurev.en.07.010162.001141

[JEB251072C22] Munz, T. (2005). The bee battles: Karl von Frisch, Adrian Wenner and the honey bee dance language controversy. *J. Hist. Biol.* 38, 535-570. 10.1007/s10739-005-0552-1

[JEB251072C23] Reynolds, A. M., Smith, A. D., Reynolds, D. R., Carreck, N. L. and Osborne, J. L. (2007). Honeybees perform optimal scale-free searching flights when attempting to locate a food source. *J. Exp. Biol.* 210, 3763-3770. 10.1242/jeb.009563 17951417

[JEB251072C24] Riley, J. R., Greggers, U., Smith, A. D., Reynolds, D. R. and Menzel, R. (2005). The flight paths of honeybees recruited by the waggle dance. *Nature* 435, 205-207. 10.1038/nature03526 15889092

[JEB251072C25] Rohrseitz, K. and Tautz, J. (1999). Honey bee dance communication: waggle run direction coded in antennal contacts? *J. Comp. Physiol. A* 184, 463-470. 10.1007/s003590050346

[JEB251072C26] Rossel, S. and Wehner, R. (1984). How bees analyse the polarization patterns in the sky: experiments and model. *J. Comp. Physiol. A* 154, 607-615. 10.1007/BF01350213

[JEB251072C27] Schmidt, I., Collett, T. S., Dillier, F. X. and Wehner, R. (1992). How desert ants cope with enforced detours on their way home. *J. Comp. Physiol. A* 171, 285-288. 10.1007/BF00223958

[JEB251072C28] Shafir, S. and Barron, A. B. (2010). Optic flow informs distance but not profitability for honeybees. *Proc. R. Soc. B* 277, 1241-1245. 10.1098/rspb.2009.1802 PMC2842809 20018787

[JEB251072C29] Si, A., Srinivasan, M. V. and Zhang, S. (2003). Honeybee navigation: properties of the visually driven odometer. *J. Exp. Biol.* 206, 1265-1273. 10.1242/jeb.00236 12624162

[JEB251072C30] Srinivasan, M. V., Zhang, S., Altwein, M. and Tautz, J. (2000). Honeybee navigation: nature and calibration of the ‘odometer’. *Science* 287, 851-853. 10.1126/science.287.5454.851 10657298

[JEB251072C31] Stone, T., Webb, B., Adden, A., Weddig, N. B., Honkanen, A., Templin, R., Wcislo, W., Scimeca, L., Warrant, E. and Heinze, S. (2017). An anatomically constrained model for path integration in the bee brain. *Curr. Biol.* 27, 3069-3085. 10.1016/j.cub.2017.08.052 28988858 PMC6196076

[JEB251072C32] Tanner, D. A. and Visscher, P. K. (2008). Do honey bees average directions in the waggle dance to determine a flight direction? *Behav. Ecol. Sociobiol.* 62, 1891-1898. 10.1007/s00265-008-0619-z

[JEB251072C33] Tautz, J. (2022). *Communication Between Honeybees: More than just a Dance in the Dark*. Springer Cham.

[JEB251072C34] Tautz, J. and Sandeman, D. C. (2003). Recruitment of honeybees to non-scented food sources. *J. Comp. Physiol. A* 189, 293-300. 10.1007/s00359-003-0402-6 12664091

[JEB251072C35] von Frisch, K. (1967). Honeybees: do they use direction and distance information provided by their dancers? *Science* 158, 1072-1076. 10.1126/science.158.3804.1072 6058349

[JEB251072C36] Wang, Z., Chen, X., Becker, F., Greggers, U., Walter, S., Werner, M., Gallistel, C. R. and Menzel, R. (2023). Honey bees infer source location from the dances of returning foragers. *Proc. Natl. Acad. Sci. USA* 120, e2213068120. 10.1073/pnas.2213068120 36917670 PMC10041085

[JEB251072C37] Wenner, A. M. (1963). The flight speed of honeybees: a quantitative approach. *J. Apic. Res.* 2, 25-32. 10.1080/00218839.1963.11100053

[JEB251072C39] Zivkovic, Z. (2004). Improved adaptive Gaussian mixture model for background subtraction. *IEEE* 2, 28-31. 10.1109/ICPR.2004.1333992

