# Supplementary material for: Honeybees express foodward flight vectors after a detour
Source: J Exp Biol. 2025 Nov 20;228(22):jeb251072. doi: 10.1242/jeb.251072 (PMC12669837; doi:10.1242/jeb.251072)
Supplement: Supplementary information [file jexbio-228-251072-s1.pdf]

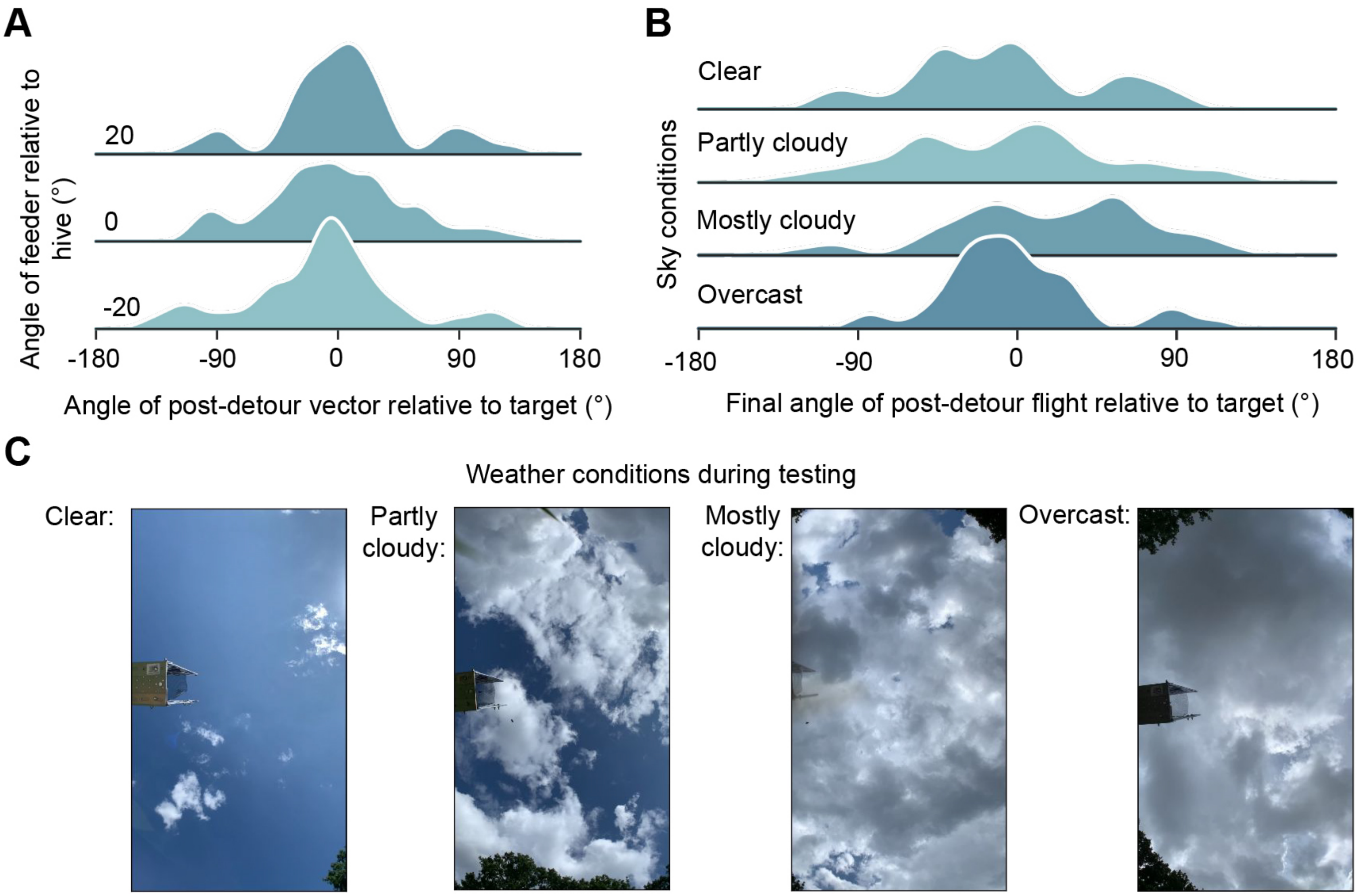

Fig. S1. Post-detour flight vectors throughout the data collection. Post-detour flight vectors across (A) different angles of the training tunnel (and thus feeder) relative to the hive and (B) sky conditions. When testing each bee, the weather condition was categorised from the video recordings as clear (*n* =40), partly cloudy (*n* =117), mostly cloudy (*n* =66) and overcast (*n* =40). The ridge plots visualise the kernel density estimates of the distributions across the given categories.

Journal of Experimental Biology • Supplementary information

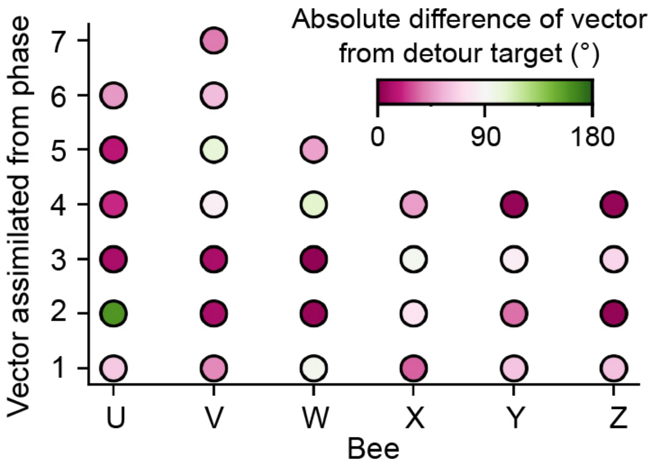

Fig. S2. No waggle phase bias in recruit's vector expression. Same recruits as in Fig. 2C but each phase they followed is coloured by the absolute difference between its angle and the final detour angle.

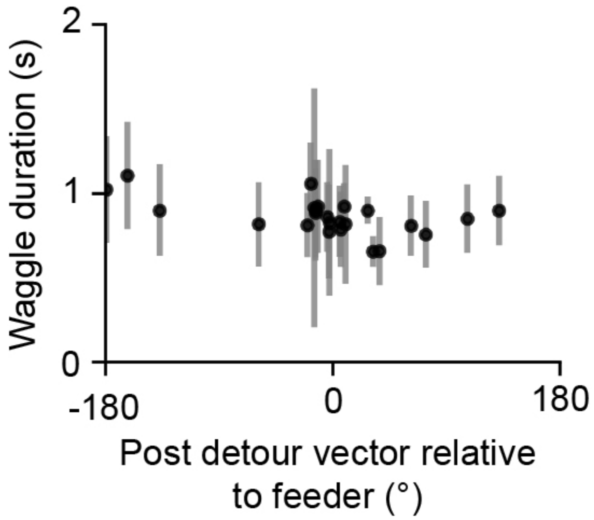

Fig. S3. Mean post-detour flight angle according to the mean ± s.d. waggle duration of the forager's dance.

Journal of Experimental Biology • Supplementary information
